# A new method for the preparation of MgAl layered double hydroxide-copper metal–organic frameworks structures: application to electrocatalytic oxidation of formaldehyde

**DOI:** 10.1038/s41598-024-55770-7

**Published:** 2024-03-03

**Authors:** Biuck Habibi, Ali Pashazadeh, Sara Pashazadeh, Lotf Ali Saghatforoush

**Affiliations:** 1 Electroanalytical Chemistry Laboratory, Department of Chemistry, Faculty of Sciences, Azarbaijan Shahid Madani University, Tabriz, 53714-161 Iran; 2 Department of Chemistry, Payame Noor University, Tehran, 19395-4697 Islamic Republic of Iran

**Keywords:** Formaldehyde, Electrocatalyst, MgAl layered double hydroxide, Copper metal–organic framework, Electrocoagulation, Carbon paste electrode, Nanoscale materials, Analytical chemistry, Catalysis, Chemical education, Electrochemistry, Energy

## Abstract

In this research, we present a novel design protocol for the in-situ synthesis of MgAl layered double hydroxide-copper metal–organic frameworks (LDH-MOFs) nanocomposite based on the electrocoagulation process and chemical method. The overall goal in this project is the primary synthesis of para-phthalic acid (PTA) intercalated MgAl-LDH with Cu (II) ions to produce the paddle-wheel like Cu-(PTA) MOFs nanocrystals on/in the MgAl-LDH structure. The physicochemical properties of final product; Cu-(PTA) MOFs/MgAl-LDH, were characterized by the surface analysis and chemical identification methods (SEM, EDX, TEM, XRD, BET, FTIR, CHN, DLS, etc.). The Cu-(PTA) MOFs/MgAl-LDH nanocomposite was used to modification of the carbon paste electrode (CPE); Cu-(PTA) MOFs/MgAl-LDH/CPE. The electrochemical performance of Cu-(PTA) MOFs/MgAl-LDH/CPE was demonstrated through the utilization of electrochemical methods. The results show a stable redox behavior of the Cu (III)/Cu (II) at the surface of Cu-(PTA) MOFs/MgAl-LDH/CPE in alkaline medium (aqueous 0.1 M NaOH electrolyte). Then, the Cu-(PTA) MOFs/MgAl-LDH/CPE was used as a new electrocatalyst toward the oxidation of formaldehyde (FA). Electrochemical data show that the Cu-(PTA) MOFs/MgAl-LDH/CPE exhibits superior electrocatalytic performance on the oxidation of FA. Also the diffusion coefficient, exchange current density (J°) and mean value of catalytic rate constant (K_cat_) were found to be 1.18 × 10^–6^ cm^2^ s^−1^, 23 mA cm^-2^ and 0.4537 × 10^4^ cm^3^ mol^−1^ s^−1^, respectively. In general, it can be said the Cu-(PTA) MOFs/MgAl-LDHs is promising candidate for applications in direct formaldehyde fuel cells.

## Introduction

Oxidation of small organic molecules has a fundamental importance in the electrocatalysis and performance of the fuel cells^1^. Formaldehyde (FA), is the simplest aldehyde which widely used as raw material in chemical and construction industry such as cosmetics, plastics and drugs^2^. Although FA is toxic and not very suitable for fuel cells, study of electrochemical oxidation of FA is important for the full understanding of methanol oxidation^3–5^. Many literatures have been explored the electrooxidation of FA on precious metal (Au, Pt, Pd, etc.) and also transition metal oxide based electrocatalysts^6,7^. However, precious metal catalysts still suffer from several problems, which need to be solved, such as their high cost, deactivation of the active sites of catalysts through the blocking by the CO poisoning^8–11^. Therefore, there is an obvious reason for finding new electrocatalysts for such important reaction. In recent years, researchers are actively working on the finding innovative electrocatalyst materials that can supplement or replace with Pt-based electrocatalysts, paving the way for more sustainable and accessible technologies^12–14^. In the investigations of electrocatalytic reactions, the discussion is often focused on the materials with high porosity and surface area^15^. Among the most familiar compounds in this field are the mesoporous and microporous materials^16^. Materials that exhibit these properties are known as metal–organic frameworks (MOFs) structures^14,17,18^. MOFs are highly crystalline subsets of microporous materials assembled by the formation of multiple coordination bonds between inorganic metal nodes and multidentate organic ligands (Linkers). These structures with incorporating specific metal centers and tailored organic linkers, can be serve as electrocatalytic platforms for various chemical reactions, including heterogeneous catalysis, photocatalysis and enzymatic-like catalysis^19–22^. During the last two decades, different synthesis methods have been developed and applied to synthesis of these materials^23^. In addition to traditional synthesis methods, new route such as mechanochemical^24^, microwave-assisted^25^, layer by layer^26^ and sonochemical have been developed for synthesis of MOFs^27^. These methods offer different advantages with few limitations, including complexity, time consumption and non-homogenous deposition. Thus, to address and resolving of these problems, designing an efficient and structured electrochemical approach, which generates desirable structures with high specific area and possible redox sites^28^. In electrochemical synthesis, neutral organic–inorganic molecules can undergo the reactions at the anode or cathode to generate anionic and cationic species, respectively^29^. These reactive intermediates can participate in various chemical transformations and leading to the formation of new compounds like as MOFs ^28,29^. It should be noted that, when MOFs are used in their pure form, good results are not obtained in electrochemistry since these materials have low intrinsic conductivity ^30^. By combining MOFs with materials that have high conductivity, the electronic conductivity of these nanoporous crystalline materials can be improved ^31,32^.

Layered double hydroxides (LDH) are ionic lamellar solids made from the stacking of positively charged (brucite-like) layers of mixed metal hydroxides, exchangeable anions and water molecules^33–36^. The exchangeable anions in LDH can be various inorganic or organic species. These anions can freely enter and exit in the interlayer regions, leading to their dynamic nature and ion-exchange capabilities. In particular, these anions have been widely used as excellent supporting materials for the growth of metal complexes in organic–inorganic systems^37,38^. The interlayer spaces of LDH can accommodate various organic molecules, providing a favorable environment for the incorporation of metal ions or complexes. This feature allows the immobilization of metal species onto the LDH surface, resulting in composite materials with enhanced properties and functionalities^39^. Therefore, using LDH as supporting materials for metal complexes opens up new avenues in various fields, including electrocatalysis, energy storage, sensors, and environmental applications^40^.The layered structure of LDH provides a unique platform for the controlled incorporation of different anions, which can alter the properties and functionalities of LDH^41,42^.

Composites of MOFs and LDH were synthesized by different synthesis routes^31^. Meanwhile, different preparation methods will introduce heterogeneous interfaces, holes and heteroatoms between LDH and MOFs to optimize the materials^43^. Recent researches have indeed reported for the synthesis of Mg–Al LDH using electrocoagulation (EC) process^44^. Electrocoagulation is one of the subfields of electrochemical economical alternative synthesis method based on the destabilization of suspended, involves the in-situ generation of coagulants by dissolution of sacrificial metal anodes with ions in solution^45^. A few studies utilized LDH-MOFs electrocatalysts prepared through electrocoagulation (EC) and chemical methods, which are known to be the best techniques to design novel electrocatalysts^46,47^. Until now, electrocatalysis of several important reaction like as: water oxidation^48,49^, oxygen evolution reaction^50,51^, water splitting^52^, rechargeable zinc–air batteries^53^, methanol oxidation^54^ and etc. have been reported on the composite materials of MOFs and LDH. With reference to the mentioned background, the aim of the present study is expanding a facile and easy approach to synthesis of the LDH supported metal ion-based MOFs nanocomposite with simple electrochemical/chemical method; in situ synthesis of the MgAl-LDH-copper metal–organic frameworks (LDH-MOFs) nanocomposite based on electrocoagulation (EC) process and chemical method. Indeed, here the primary synthesis of para-phthalic acid (PTA) intercalated MgAl-LDH with Cu (II) ions to produce the paddle-wheel like Cu-(PTA) MOFs nanocrystals on/in the MgAl-LDH structure is going happen. The as-synthesized nanocomposite; Cu-(PTA) MOFs/MgAl-LDH, was characterized by several techniques, including SEM, EDX, TEM, PSD, BET, TGA, XRD, FT-IR, DLS, and etc. Then, a comprehensive understanding of the electrochemical activity of the Cu-(PTA) MOFs/MgAl-LDH/CPE was evaluated for the first time toward the oxidation of FA by employing the electrochemical techniques (such as cyclic voltammetry, chronoamperometry and pseudo steady-state polarization) in alkaline media. The results showed that the prepared modified electrode had good electrocatalytic properties toward FA electrooxidation. Indeed, this work provides a good idea for the design of non-precious metal electrocatalysts with the high performance.

## Experimental

### Materials and instruments

The chemicals were used in this research including: Sodium chloride (NaCl) (99%), para-phthalic acid (PTA), copper (II) nitrate trihydrate [Cu(NO_3_)_2_. 3H_2_O], ethanol, dimethylformamide, formaldehyde (CH_2_O), sodium hydroxide (NaOH) purchased from commercial sources and used without further purification. The aluminum and magnesium plates were purchased from a reputable company. The solutions were made with double distilled water. The surface morphology and structural of the prepared materials was evaluated by using a scanning electron microscopy (SEM), energy dispersive x-ray spectroscopy (EDX) (MIRA3 FEG-Tescan) and transmission electron microscopy (TEM) (Carl Ziess AG-Zeiss EM900). The X-ray diffraction (XRD) patterns were collected within the range of 5.3–80° 2θ on a Bruker D_8_ advance diffractometer with Cu K radiation (λ = 0.154056 nm). Thermogravimetric analysis (TGA) was performed using SETARAM SETSYS 16/18 thermal analyzer heating instruments (heating rate of 5 °C/min) in the nitrogen flow atmosphere (25 mL per min). Specific surface area and pore size distribution of the samples was determined by analyzing N_2_ physisorption isotherms (BELSORP Mini II). For determination of percentage C, N and H, the CHN analysis was carried out by Euro EA elemental analyzer. Particle sizes of the synthesized materials were recorded by using a particle size analyzer (ZEN3600, United Kingdom) in respective aqueous suspensions. All the voltammetric measurements were carried out using an Autolab potentiostat/galvanostat PGSTAT 100 equipped with a 3-electrode containing modified or unmodified CPE as working electrode, a platinum wire as a counter (auxiliary) electrode and silver/silver chloride (Ag/AgCl) as a reference electrode at room temperature. All voltammetric data were transformed to EXCEL files (version19).

### Preparation of the MgAl-(PTA) LDH and Cu-(PTA) MOFs/MgAl-LDH through the electrocoagulation and chemical method

The MgAl-LDH was prepared using a simple electrocoagulation (EC) method^55^. For this purpose, a reactor with the following characteristics was used for experiments: Electrocoagulation experiments were conducted in a batch mode, in a 1 L glass reactor using parallel aluminum (Al) and magnesium (Mg) plate electrodes. The area of each metal plate was 20 cm^2^, which connected to the power supply which provide the necessary voltage for the electrocoagulation process. The Al electrode was connected to the positive pole of the power supply and the Mg electrode was connected to the negative pole of the power supply. Sodium chloride solution (solution A) was used to regulate the electrical conductivity. In the next step, a 10 mM solution containing sodium hydroxide and terephthalic acid was prepared and named as solution B. Then, while the cell containing sodium chloride (solution A) is stirred on a magnetic stirrer and a 5 mA current is applied between the Al and Mg electrodes, solution B was slowly added into the solution A. The resulting electrochemical reaction produces a white precipitate. The resulting MgAl-(PTA) LDH precipitate was filtered through quantitative filter paper (Grade 41) and washed with double distilled water 2 times. In the following, the Cu-(PTA) MOFs/MgAl-LDH was prepared by using 3 g MgAl-(PTA) LDH and 2.172 g Cu (NO_3_)_2_. 3H_2_O dispersed in 50 mL DMF. The mixture was refluxed at (110–120) °C for 1 h. A green color product was obtained. The obtained product was washed with double distilled water and ethanol and dried over vacuum desiccator.

### Procedure for the preparation of unmodified and modified CPE

**Figure 1:**
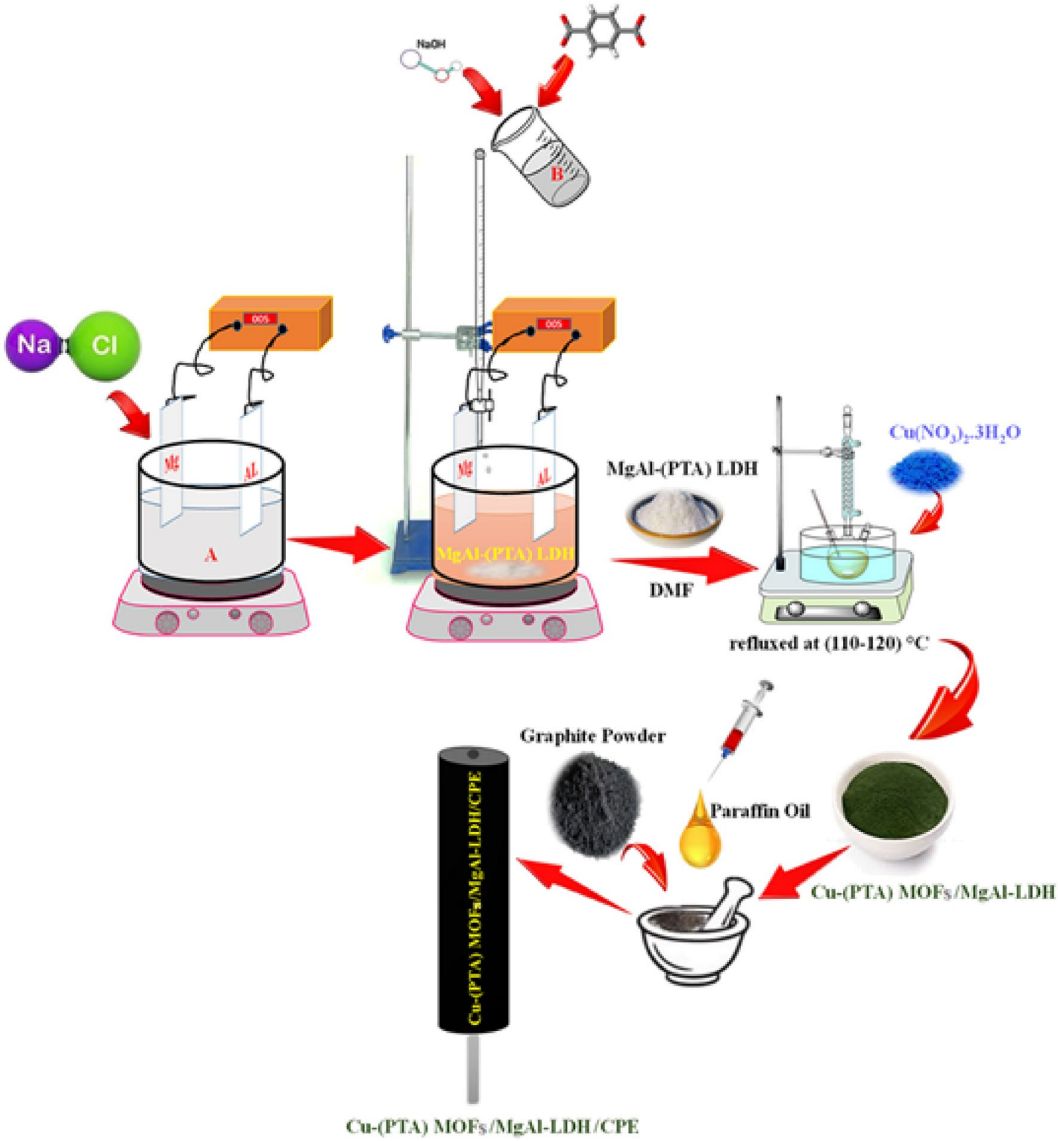
Schematic preparation steps of the Cu-(PTA) MOFs/MgAl-LDH/CPE.

In this work the preparation of unmodified CPE is as the same as which described in our previous work^56^. The mixing of the graphite powder and liquid paraffin was done by means of a pestle and a mortar in order to obtain a homogeneous paste, which was used to fill the working electrode hole. Hole filling is made in small portions when each of them being pressed intimately before adding the next one. Then the CPE was smoothed onto a white, clean and soft paper in order to remove the excess of carbon paste. The electrical contact was made via copper wire inserted into the syringe and into the back of the composite past. CPE lefts unused for a certain time (15 h) to allow their final homogenization to proceed. This process of "self-homogenization" has been confirmed experimentally; freshly homogenized CPEs often exhibit a rather unstable behavior. For the preparation of the modified CPE, the Cu-(PTA) MOFs/MgAl-LDH was grounded with graphite powder with the ratio 3:70 (w/w) for achieving a uniformly wetted paste and then the paste was packed into working electrode hole (ca.3.4 mm i.d. and 10 cm long) and pressed thoroughly by mechanical force. Then, the excess paste, if present, remove carefully and smoothing the surface on a weighing paper^57^. The schematic preparation steps of the Cu-(PTA) MOFs/MgAl-LDH/CPE was presented in Fig. 1. As above, modified CPEs left unused for 15 h to allow their final homogenization to proceed and dried.

## Results and discussion

### Physicochemical characterization of synthesized materials

**Figure 2:**
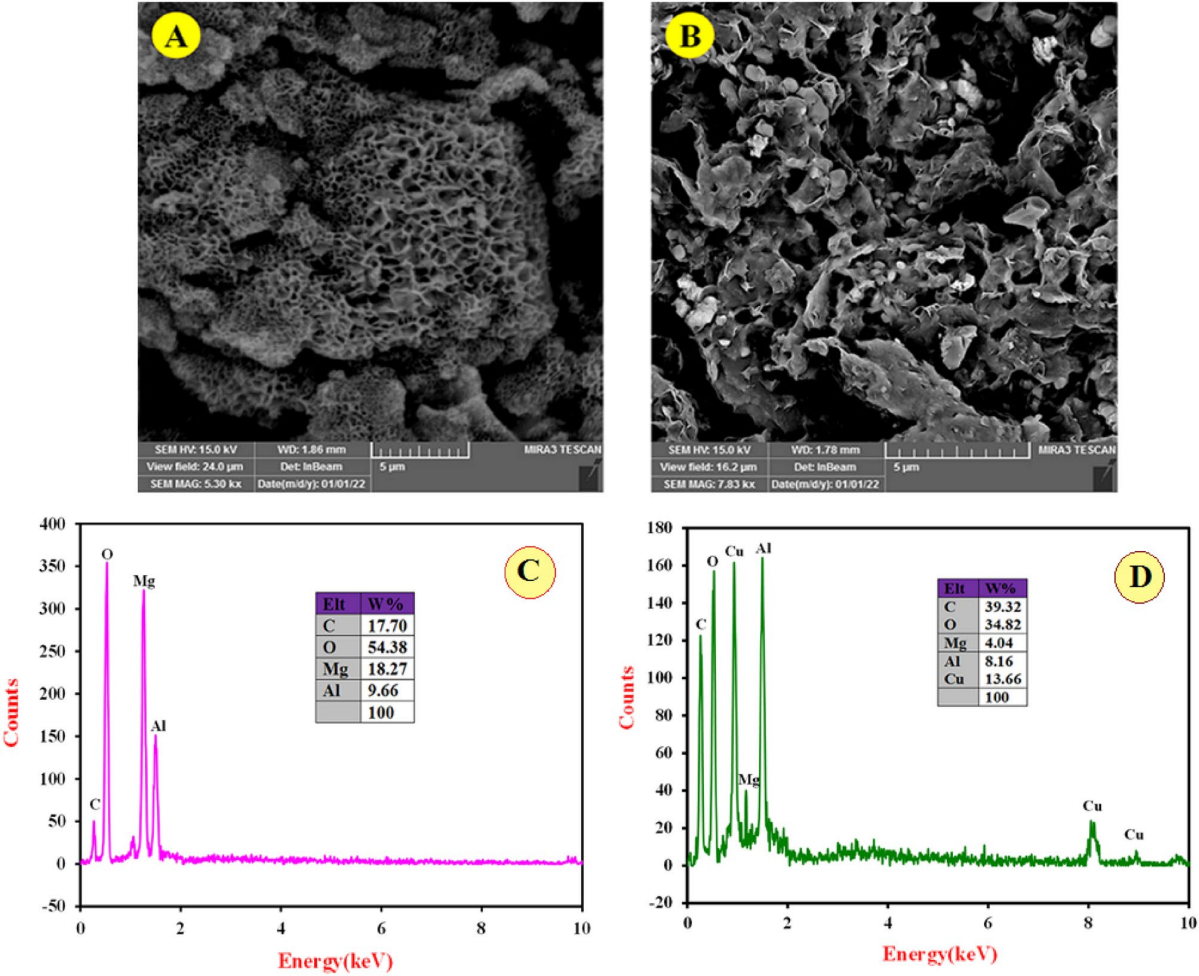
SEM images of (**A**) MgAl-(PTA) LDH and (**B**) Cu-(PTA) MOFs/MgAl-LDH. EDX spectra of the (**C**) MgAl-(PTA) LDH and (**D**) Cu-(PTA) MOFs/MgAl-LDH.

**Figure 3:**
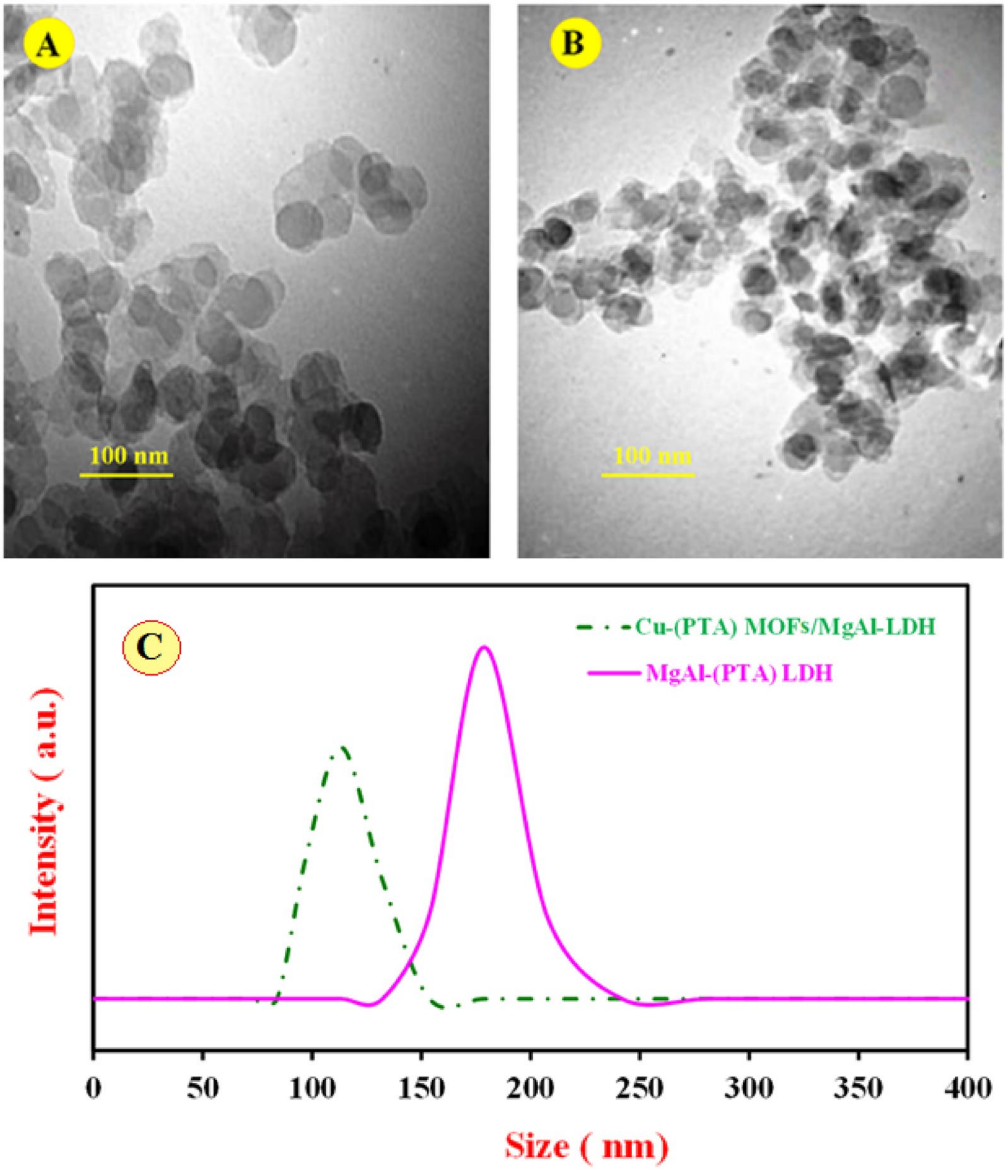
TEM images of (**A**) MgAl-(PTA) LDH and (**B**) Cu-(PTA) MOFs/MgAl-LDH. (**C**) Particle size distribution of the MgAl-(PTA) LDH (red continuous line) and Cu-(PTA) MOF/MgAl-LDH (green dashed line) in respective aqueous suspensions.

**Figure 4:**
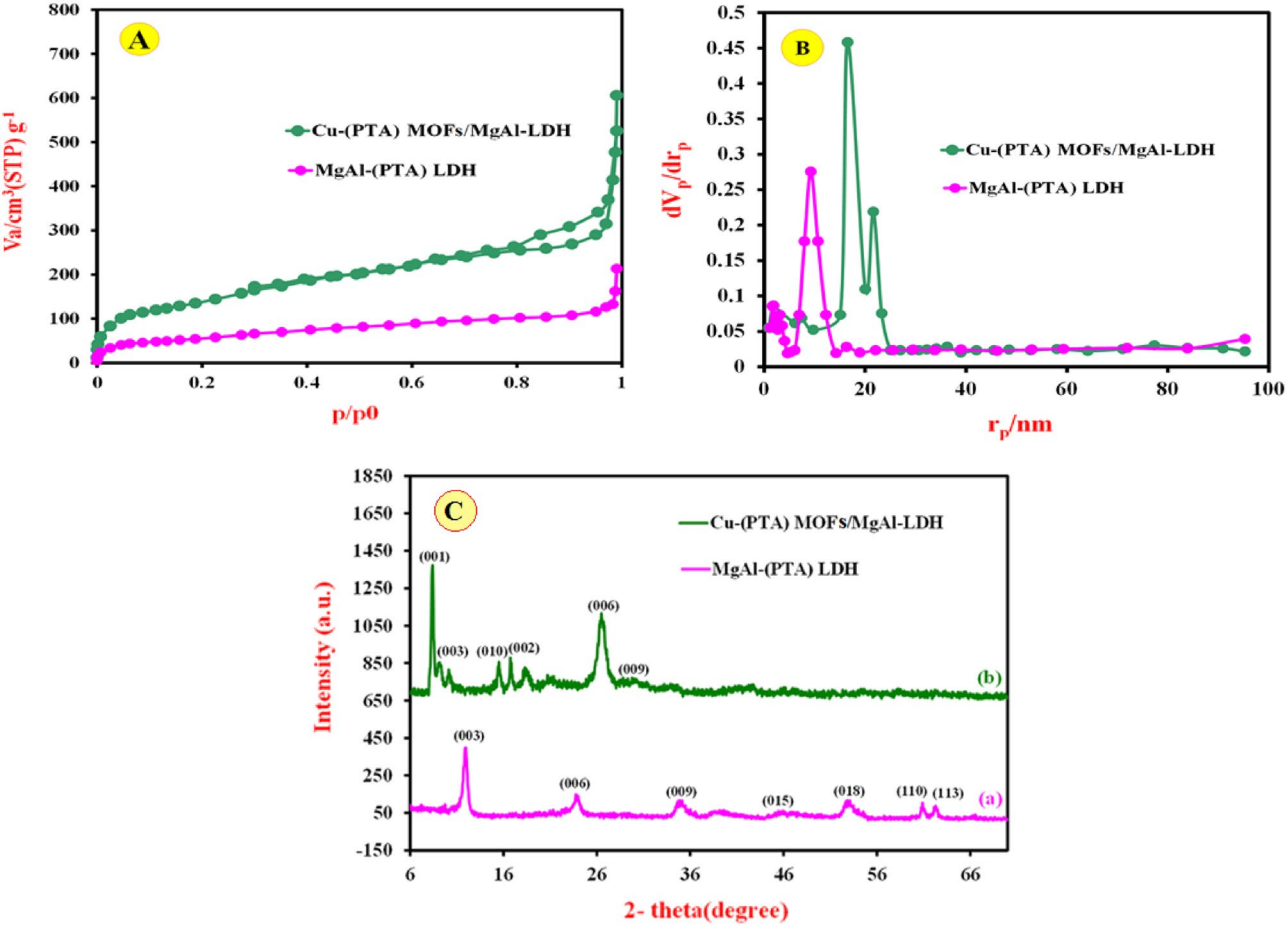
N_2_ adsorption–desorption isotherms of the MgAl-(PTA) LDH and Cu-(PTA) MOFs/MgAl-LDH (**A**) and pore size distributions of the MgAl-(PTA) LDH and Cu-(PTA) MOFs/MgAl-LDH (**B**). (**C**) XRD patterns of MgAl-(PTA) LDH (**a**) and Cu-(PTA) MOFs/MgAl-LDH (**b**).

**Figure 5:**
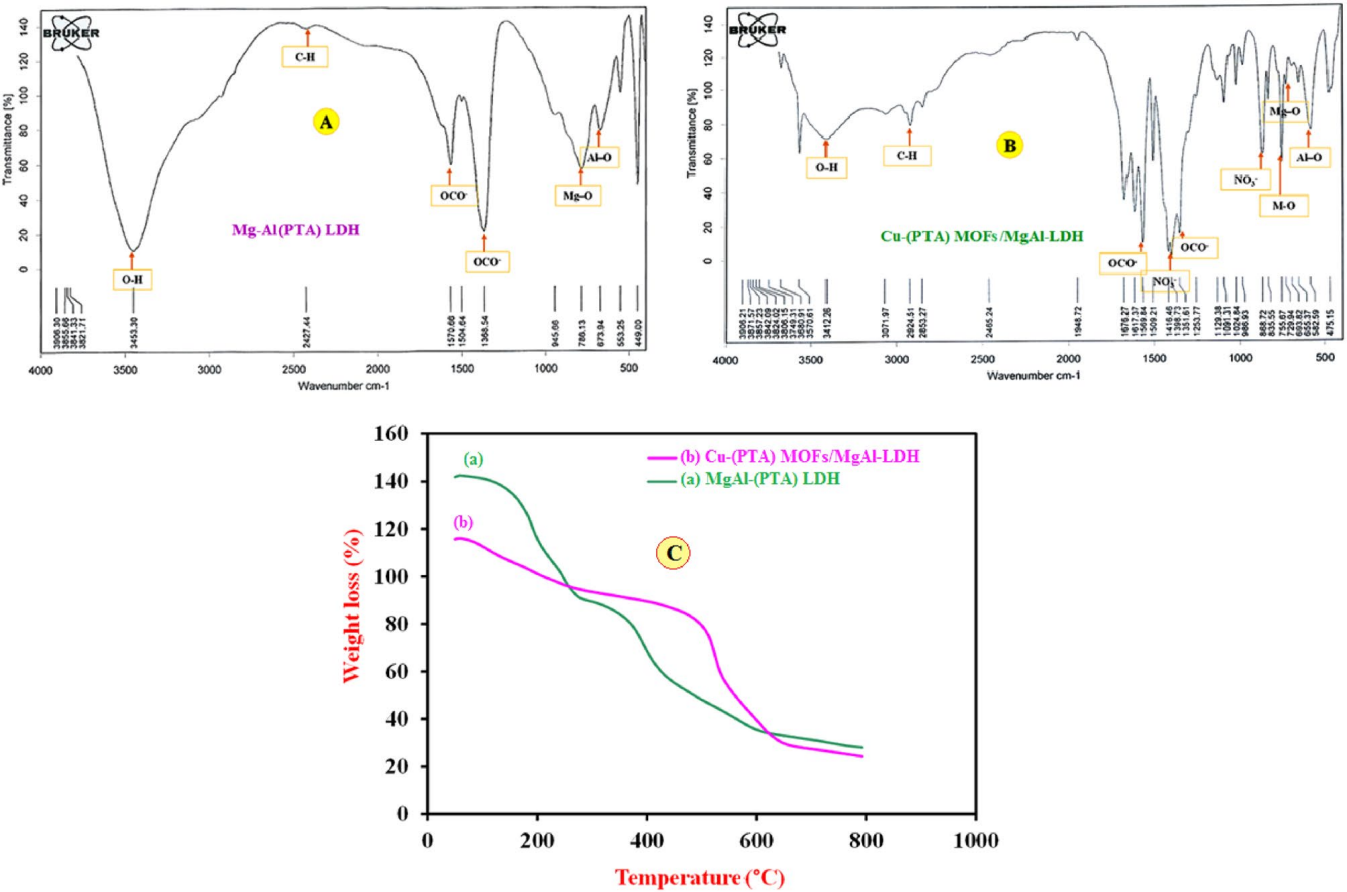
FT-IR spectra of (**A**) MgAl-(PTA) LDH and (**B**) Cu-(PTA) MOFs/MgAl-LDH. (**C**) TGA curves of the MgAl-(PTA) LDH (**a**) and Cu-(PTA) MOFs/MgAl-LDH (**b**).

The surface morphology, elemental composition, internal and crystal structure of the synthesized materials were analyzed by a variety of diagnostic tools to obtain the morphological, structural and elemental information about the resulted nanocomposite. Typical morphology of the synthesized MgAl-(PTA) LDH and Cu-(PTA) MOFs/MgAl-LDH was depicted by SEM and shown in Fig. 2A,B. The captured image A exhibits aggregated and a flake-like morphology. Figure 2B shows the SEM image of the Cu-(PTA) MOFs/MgAl-LDH with a distribution of uniforms microcrystals of Cu-(PTA) MOFs/MgAl-LDH composition. It seems that hydroxide groups existence interlayer of LDH, regulated formation of Cu-(PTA) MOFs particles through mechanism-oriented growth on MgAl-(PTA) LDH surface^58,59^. Furthermore, the spectra related to elemental analysis (EDX) of MgAl-(PTA) LDH and Cu-(PTA) MOFs/MgAl-LDH are also shown in Fig. 2C,D. In Fig. 2C, the EDX spectrum of MgAl-(PTA) LDH shows the strong peaks of C, O, Mg and Al with weight percentages of 17.70, 54.38, 18.27 and 9.66%, respectively. Figure 2D refers to EDX spectrum of the Cu-(PTA) MOFs/MgAl-LDH which represents the growth of copper-MOFs on/in MgAl-(PTA) LDH with strong peaks of C, O, Mg, Al and Cu with weight percentages of 39.32, 34.82, 4.04, 8.16 and 13.66%, respectively. For examination of the topography of internal structure, TEM images of the MgAl-(PTA) LDH and Cu-(PTA) MOFs/MgAl-LDH were prepared and shown in Fig. 3A,B. It is quite clear that, the translucent plate-like morphology could be clearly in MgAl-(PTA) LDH (Fig. 3A) with a size about 30–50 nm arising from insertion of PTA particles between LDH layers and the change in the structure of the LDH plates. In the second stage, by introducing copper into the interlayer structure of LDH and creating a new structure (Cu-(PTA) MOFs), it can be observed (Fig. 3B) that a series of dense structures were created with a deformed hexagonal structure and the most of the particles are normal in size (20–50 nm). The particle size distribution of the MgAl-(PTA) LDH and Cu-(PTA) MOFs/MgAl-LDH in respective aqueous suspensions was analyzed by dynamic light scattering (DLS) technique and presented in Fig. 3C. The particle sized results of the MgAl-(PTA) LDH (red continuous line), with size ranged between 113 and 400 nm and mean particle size about of 175 nm. In Fig. 3C, green dashed line shows the DLS result of the Cu-(PTA) MOFs/MgAl-LDH with particle size which was determined between 70 and 150 nm, and mean particle size of 105 nm. The decrease in particle size of Cu-(PTA) MOFs/MgAl-LDH vs MgAl-(PTA) LDH due to the entry of copper ions and formation of MOFs structure is in good agreement with the results of the TEM analysis. In the following, the fundamental physicochemical properties (specific surface area and pore-size distribution) of the MgAl-(PTA) LDH and Cu-(PTA) MOFs/MgAl-LDH were studied from N_2_ adsorption–desorption plots and the Barret-Joyner-Halenda (BJH) curve with physisorption isotherms as shown in Fig. 4 A and B, respectively. The samples displayed a type IV isotherm with H3 hysteresis loops and a type IV isotherm with sharp uptakes and H_3_-type hysteresis loops. In terms of experimental research, the most important data obtained from the analysis of Brunauer–Emmett–Teller (BET) curve: specific surface area and the average pore size distribution. In this case, the BET specific surface area of the MgAl-(PTA) LDH was found to be 137 m^2^/g, while for the Cu-(PTA) MOFs/MgAl-LDH, it was 111 m^2^/g. Moreover, the average pore size distribution of the MgAl-(PTA) LDH was determined by BHJ plot as 18 nm, while pore size of the Cu-(PTA) MOFs/MgAl-LDH was obtained as ≈10 nm that the sample was composed of hierarchical porous material. Therefore, the formation of the Cu-(PTA) MOFs composite within MgAl-(PTA) LDH leads to the reduction of the cavity space in the interlayer of LDH, which results in a decrease in the surface area and volume of cavity of the final compounds. In addition, the reduction of the average diameter of the cavity from 18 nm for MgAl-(PTA) LDH to 10 nm for Cu-(PTA) MOFs/MgAl-LDH is evidence of the functionalization of the holes of the MgAl-(PTA) LDH^60,61^. In order to examine the crystal structure of the synthesized materials, the XRD technique was employed and results shown in Fig. 4C. The patterns exhibited five peaks at 2θ of 12.36, 24.13, 35.2, 46, 53.23, 62.12 and 62.96 corresponding to (113), (110), (018), (015), (009), (006) and (003) planes of MgAl-(PTA) LDH (pattern a)^44^. In addition, XRD pattern of the Cu-(PTA) MOFs/MgAl-LDH (pattern b) in Fig. 4C contains six main peaks in the 2θ of 8.44°, 10.36°, 15.56°, 16.76°, 26.72°and 30.56° corresponding to planes of (001), (003), (010), (002), (006) and (009), respectively^45^. Therefore, in general, it can be said that the Bragg angles in the XRD pattern of Cu-(PTA) MOFs/MgAl-LDH are shifted to lower angles than the peaks of MgAl-(PTA) LDH, which indicates an increase in the distance between layers of MgAl-(PTA) LDH and the formation of Cu-MOFs particles in these spaces^62,63^. Also, to study and identify the functional groups in MgAl-(PTA) LDH and Cu-(PTA) MOFs/MgAl-LDH, the Fourier transform infrared spectroscopy (FT-IR) technique was used. According to the obtained results in Fig. 5A, the peaks in the wavelength number of 673 and 786 cm^-1^ are related to the stretching vibrations of Al-O and Mg-O functional groups^64,65^. Furthermore, the broad and strong peak at the wavelength number 3453 cm^-1^ is related to the O–H bond, which indicates the presence of water molecules in the between layers in brucite layers of MgAl-(PTA) LDH^66^. Also, the peaks at 1368, 1570, 1351 and 673 cm^-1^ are attributed to the symmetric and asymmetric stretching states of the carboxylate group (OCO^–^), stretching vibrations of the carboxylate group (OCO^–^) and C–H vibrational modes in MgAl-(PTA) LDH^67^. In addition, the peaks in 1416 and 1569 cm^-1^ in Fig. 5B are attributed to the coordination of PTA ligand to Cu (II) ion in Cu-(PTA) MOFs/MgAl-LDH composite^68^. The two sharp and strong peaks observed at the 868 and 1398 cm^-1^ in the resulting spectrum of Cu-(PTA) MOFs/MgAl-LDH are related to the stretching vibrations of the interlayer nitrate anions and confirm the fact that the nitrate group in the LDH interlayer plates with bonds MOFs have been replaced. Also, the peak observed at the wavelength number of 755 cm^-1^ is related to the lattice vibrations of M–O and M–O–M bonds in the octahedral planes of LDH-MOF bonds in the synthetic Cu-(PTA) MOFs/MgAl-LDH which is close to similar reported papers^47,68^. The CHN analyses of MgAl-(PTA)LDH and Cu-(PTA) MOFs/MgAl-LDH nanocomposite are given in Table 1. Based on the results, the carbon content for the Cu-(PTA) MOFs/MgAl-LDH has increased from 31.713 to 37.668% after the Cu-MOFs formation and intercalated into the interlamellar gallery. Additionally, the content of nitrogen in the MgAl-(PTA) LDH after the intercalation process has significantly increasing from the 2.461 to 7.502%, implying that the nitrate ions have been exchanged with linker onions in the MgAl-(PTA) LDH due to reaction PTA with Cu cations introduce on the interlamellar gallery of LDH compound^69^. The appearance of two sharp and strong peaks in the resulting spectrum of Cu-(PTA) MOFs/MgAl-LDH in FT-IR (Fig. 5B) is related to the interlayer nitrate anions confirm this phenomenon and is in good agreement with the results of the CHN analysis results.

**Table 1:** CHN analysis of MgAl-(PTA) LDH and Cu-(PTA) MOFs/MgAl-LDH nanopowders.

Element	Content (%)	
	MgAl-(PTA)LDH	Cu-(PTA)MOF/MgAl-LDH
C	31.713	37.668
N	2.461	7.502
H	3.359	4.785

Thermal characterization of the obtained materials has been made using TGA, as depicted in Fig. 5C. Curve a, the TGA result for MgAl-(PTA) LDH, displays three main stages of weight loss. The initial weight loss at around 73 °C is attributed to the removal of loosely bound water molecules from the LDHs interlayer. The second weight loss, occurring in the temperature range of 267–485 °C, leads to the decomposition of PTA in the interlayer structures. The third and final weight loss, observed in the temperature range of 470–635 °C, is likely due to dehydroxylation and decarbonation of the LDH sheets to form mixed solid oxides^70^. A similar interpretation can be made about the composition of Cu-(PTA) MOFs/MgAl-LDH, curve b in Fig. 5C. The second and third features in the Cu-(PTA) MOFs/MgAl-LDH overlap due to the decomposition of the Cu-(PTA) MOFs/MgAl-LDH. The framework decomposition of Cu-(PTA) MOFs significantly impacts the thermal decomposition process and results in the complete collapse of the material's structure. Additionally, the increased thermal stability in Cu-(PTA) MOFs/MgAl-LDH up to 500 °C indicates a strong interaction between MOF and LDH sheets compared to the organic PTA intercalated MgAl-LDH.

### Electrochemical characteristics of the Cu-(PTA) MOFs/MgAl-LDH

#### Electrochemical behavior of the Cu-(PTA) MOFs/MgAl-LDH in alkaline media

**Figure 6:**
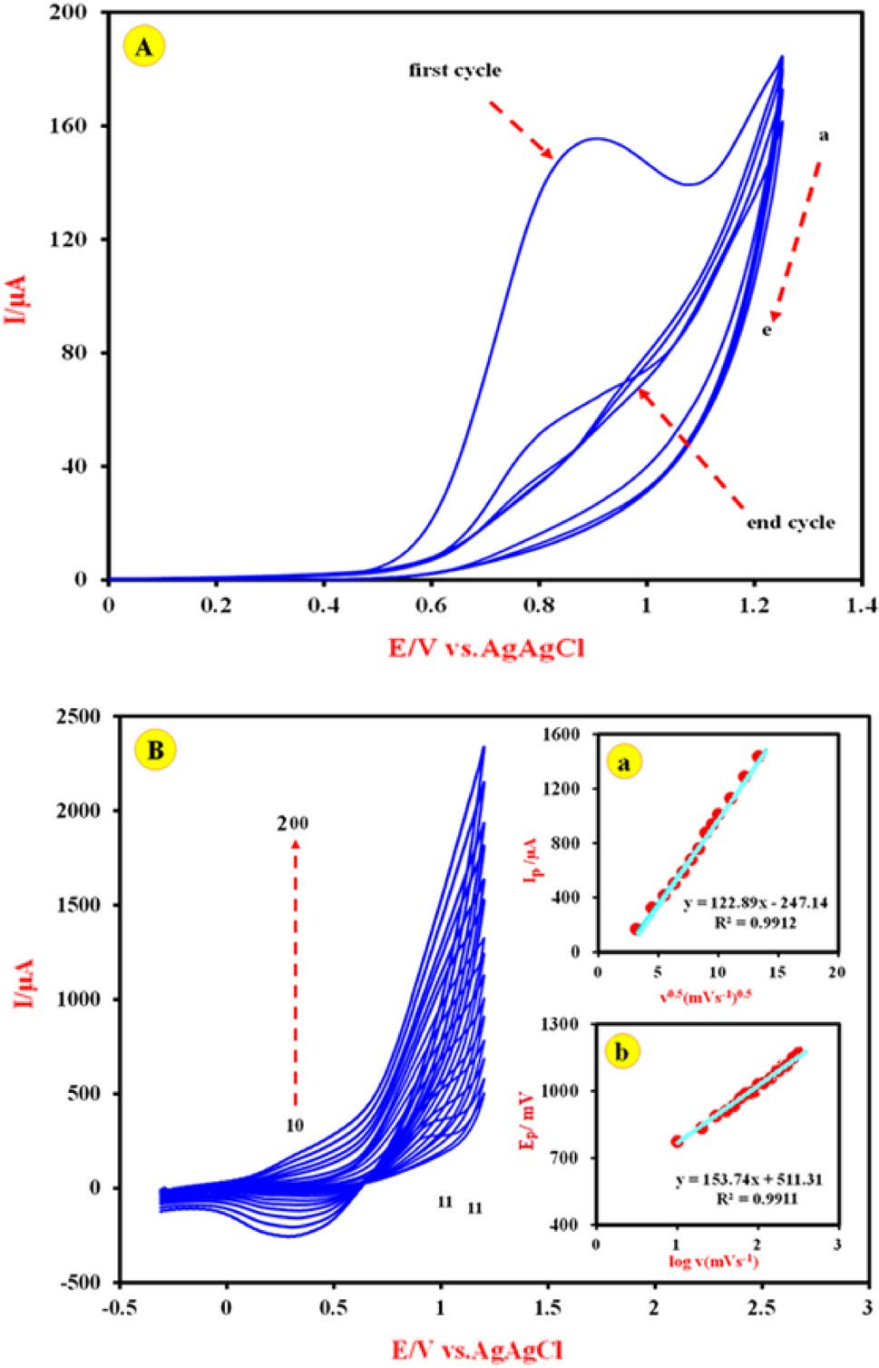
(**A**) Repetitive cyclic voltammograms of Cu-(PTA) MOFs/MgAl-LDH/CPE in 0.1 M NaOH in the potential range of 0–1200 mV. Potential scan rate is 50 mV s^-1^. (**a**) First cycle; (**e**) 5th cycle. (**B**) Cyclic voltammetric curves of Cu-(PTA) MOFs/MgAl-LDH/CPE in 0.1 M NaOH at various potential scan rates 10, 20, 30, 40, 50, 60, 70, 80, 90, 100, 150, 160, 170, 190, and 200 mVs^-1^. Inset (**a**): The dependency of anodic peak currents vs υ^1/2^. Inset (**b**): The plot of E_p_ vs. log υ.

Initially, for the activation of the Cu-(PTA) MOFs/MgAl-LDH/CPE, its cyclic voltammograms (CVs) (five cycles) were recorded in appropriate range of potential from 0.0 to 1200 mV vs. Ag/AgCl in 0.1 M NaOH at a scan rate of 50 mV s^-1^ and results shown in Fig. 6A. The CVs show that the appearance of the voltammograms changes during the number of cycles for the modified electrode. As can be seen, in the first scan, an anodic peak appears in the area of formation of active copper species. In the next cycles, the current of anodic peak is reduced, so that after five cycles, it reaches almost to zero current with an irreversible behavior. Therefore, it can be said that, during successive cycling, the oxidation reaction starts with the interaction between the copper (II) ions present in the Cu-(PTA) MOFs/MgAl-LDH/CPE and the sodium ions in the solution according to the following reaction^57^:

1 $${\text{Cu}}\left({\text{II}}\right)[{\text{MOFs}}/{\text{MgAl}}-{\text{LDH}}]+ 2{{\text{Na}}}_{\left({\text{s}}\right)}^{+}\to {{\text{Cu}}({\text{II}})}_{({\text{interface}})} + {{Na}^{+}}_{(MOFs-LDH)}$$

As stated by Eq. (1), the electroactive ions, Cu (II) ions at the modified electrode surface are oxidized to Cu (III) species. In the next cycles, due to the entry of OH^-^ ions into the MOFs/MgAl-LDH composite structure and the conversion of Cu (II) to Cu (OH)_2_ and Cu (OH)_2_ to Cu (III), were take place at the surface of modified electrode according to the following reaction^71,72^:

2 $$2{\text{Cu}}\left({\text{II}}\right){\text{MOFs}}/{\text{MgAl}}-{\text{LDH}}+ 2{{\text{OH}}}^{-}\to \mathrm{ Cu}{\left({\text{OH}}\right)}_{2}{\text{MOFs}}/{\text{MgAl}}-{\text{LDH}}+ 2{{\text{e}}}^{-}$$

3 $${\text{Cu}}{\left({\text{OH}}\right)}_{2}{\text{MOFs}}/{\text{MgAl}}-{\text{LDH}}+ 2{{\text{OH}}}^{-}\to {\text{Cu}}\left({\text{III}}\right){\text{OOHMOFs}}/{\text{MgAl}}-{\text{LDH}}+{{\text{H}}}_{2}{\text{O}}+{{\text{e}}}^{-}$$

Figure 6B illustrates the typical CVs of the Cu-(PTA) MOFs/MgAl-LDH/CPE at various scan rates (10–200 mV s^-1^) in 0.1 M NaOH solution. The results show that the anodic peak currents are proportional with the scan rate. The obtained curve, inset a, in Fig. 6B confirms the liner dependence of I_p_ versus ν^1/2^, which indicates a diffusion-controlled process^73^. Also, the transfer coefficient was calculated from the plot of peak potentials (E_p_) vs logarithm of scan rate (Fig. 6B inset b) and Eq. (4) as α = 0.19.

4 $${E}_{p}=k+\left(\frac{2.3{\text{RT}}}{\left(1-{\mathrm{\alpha }}_{{\text{s}}}\right){\text{nF}}}\right){\text{log}}\left(v\right)$$

The surface coverage (Г*) (normalized to the geometric area) of the active spices at the Cu-(PTA) MOFs/MgAl-LDH/CPE was measured from the slope of anodic peak current (I_p_) vs scan rate as the 7.72 × 10^–5^ mol cm^-2^ for n = 1:

5 $${{\text{I}}}_{{\text{p}}}={({\text{n}}}^{2}{F}^{2}/4{\text{RT}}){\mathrm{\nu A \Gamma^{*}}}$$

In Eq. 5, A is the working electrode area, ν is the scan rate and Γ* is the surface coverage of the active species^74^.

To obtain the electroactive surface area of the Cu-(PTA) MOFs/MgAl-LDH/CPE, the [Fe(CN)_6_]^−3/−4^ ions (5 mM) was used as a probe redox system. According to the Randles–Sevcik equation (Eq. 6):

6 $${I}_{p}=2.69\times {10}^{5}A{D}^{1/2}{n}^{3/2}C{v}^{1/2}$$

where: (I_p_) is the peak current, (n) is the number of electrons transferred in the reaction, (D) is the diffusion coefficient and C is the concentration of [Fe(CN)_6_]^−3/−4^, (v) is the scan rate (V/s) and A is the active surface area of the modified electrode (cm^2^). The CVs of the Cu-(PTA) MOFs/MgAl-LDH/CPE were recorded in different scan rate (not shown here) and the results were analyzed; I_p_ vs ν^1/2^. From the slope, the active surface areas of the Cu-(PTA) MOFs/MgAl-LDH/CPE was determined as 0.46 cm^2^.

#### Electrocatalytic activity of the Cu-(PTA) MOFs/MgAl-LDH/CPE toward FA oxidation

**Figure 7:**
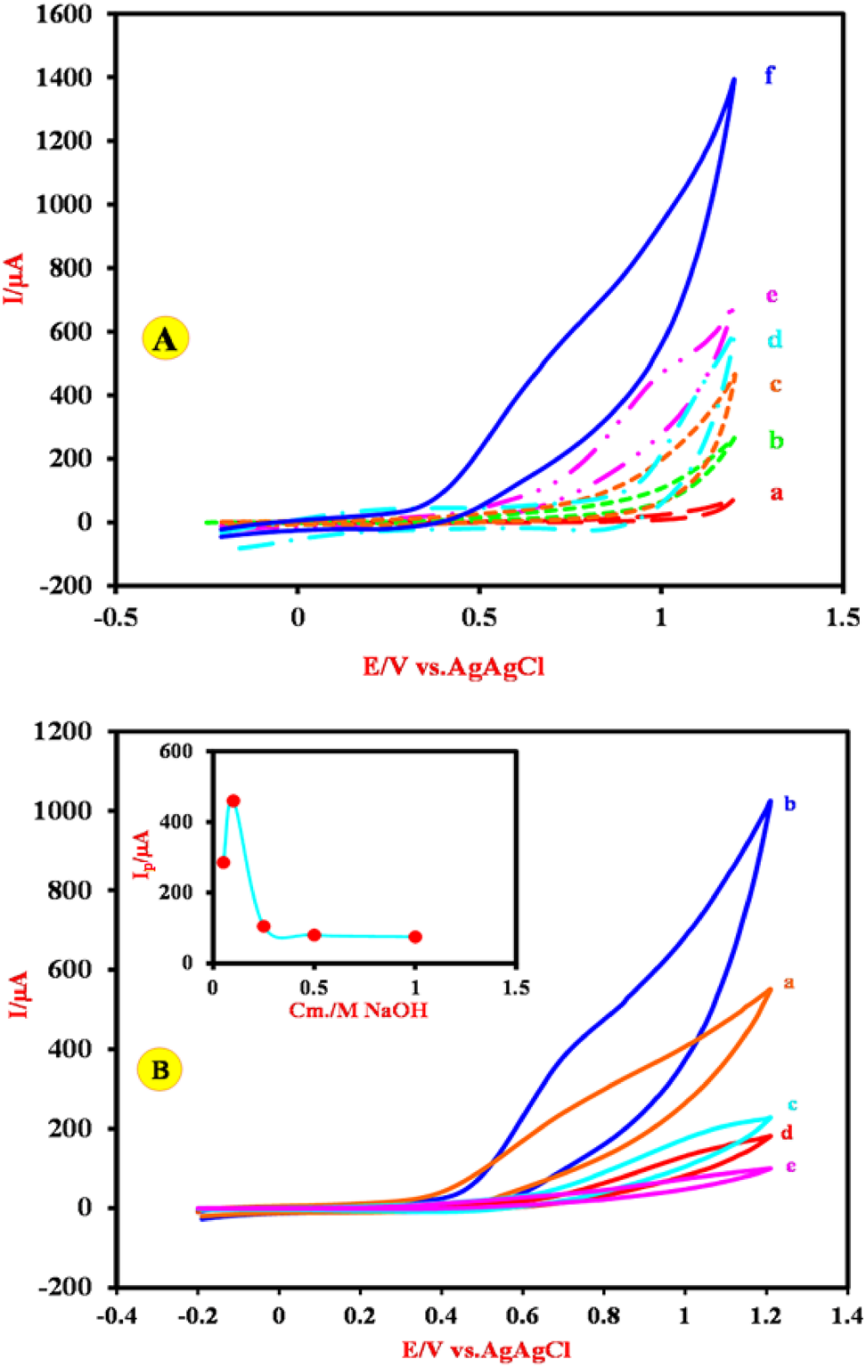
(**A**) Cyclic voltammograms of CPE (a and b curves), MgAl-(PTA) LDH/CPE (c and d curves), Cu-(PTA) MOFs/MgAl-LDH/CPE (e and f curves) in 0.1 M NaOH solution in the absence and presence of 66 mM FA at the scan rate of 50 mV s^−1^, respectively. (**B**) Cyclic voltammograms of the Cu-(PTA) MOFs/MgAl-LDH/CPE in different concentrations of NaOH in the presence of 66 mM FA with a scan rate of 50 mV s-^1^. Inset: Anodic peak currents vs NaOH concentrations.

In following, to investigate the electrocatalytic activity of the Cu-(PTA) MOFs/MgAl-LDH/CPE toward the oxidation of FA, the CVs of unmodified carbon paste (UCPE), MgAl-(PTA) LDH/CPE and Cu-(PTA) MOFs/MgAl-LDH were recorded in 0.1 M NaOH solution as background electrolyte in the absence and presence of 66 mM FA and shown in Fig. 7A. As can be seen, there are no obvious redox peaks (anodic and cathodic peaks) on the voltammograms of the UCPE in 0.1 M NaOH solution without FA (curve a) and also after the addition of FA (curve b). Therefore, the UCPE is electrochemically inactive for FA oxidation. Figure 7A curve c shows the electrochemical behavior of MgAl-(PTA) LDH/CPE in the background electrolyte and curve d shows the same electrode in the presence of FA. Indeed, by modifying the CPE with MgAl-(PTA) LDH, the obtained electrode does not have a favorable electrocatalytic behavior toward the oxidation of FA. On the other hand, by inserting of the copper ions to the MgAl-(PTA)LDH/CPE and construction of the Cu-(PTA) MOFs/MgAl-LDH/CPE, the amount of anodic peak current is increased in the presence of FA (curve f) (curve e shows the CV of the same electrode in the background electrolyte) which indicates that the incorporation of Cu (II) into the MgAl-(PTA)LDH and preparation of the Cu-(PTA) MOFs/MgAl-LDH/CPE leading the oxidation of Cu (II) to Cu (III) and electrocatalysis of the FA oxidation and appearance of a high oxidation peak current, which represents the electrocatalytic behavior of the Cu-(PTA) MOFs/MgAl-LDH/CPE toward the oxidation of FA^75,76^:

7 $${\text{Cu}}\left({\text{III}}\right){\text{OOHMOFs}}-{\text{LDH}}+{{\text{CH}}}_{2}\mathrm{O }({\text{FA}})\to {\text{Cu}}\left({\text{II}}\right){\text{MOFs}}-{\text{LDH}}+\mathrm{oxidation\, producs}$$

In fact, it can be suggested that the bonded copper ions in the MgAl-(PTA)LDH structure act as intermediate ions in the FA electrooxidation reaction^77^. On the other hand, the redox Cu couple Cu(II)/Cu(III) has play as a mediator role on heterogeneous catalytic oxidation of FA. Indeed, FA is firstly oxidized to format ions in alkaline medium, and then the format ions are oxidized to carbon dioxide, Eq. (8) ^77^.

8 $$HCOH\stackrel{-2{e}^{-}+2O{H}^{-}}{\longrightarrow }HCO{O}^{-}\stackrel{-2{e}^{-}+O{H}^{-}}{\longrightarrow }C{O}_{2}$$

**Figure 8:**
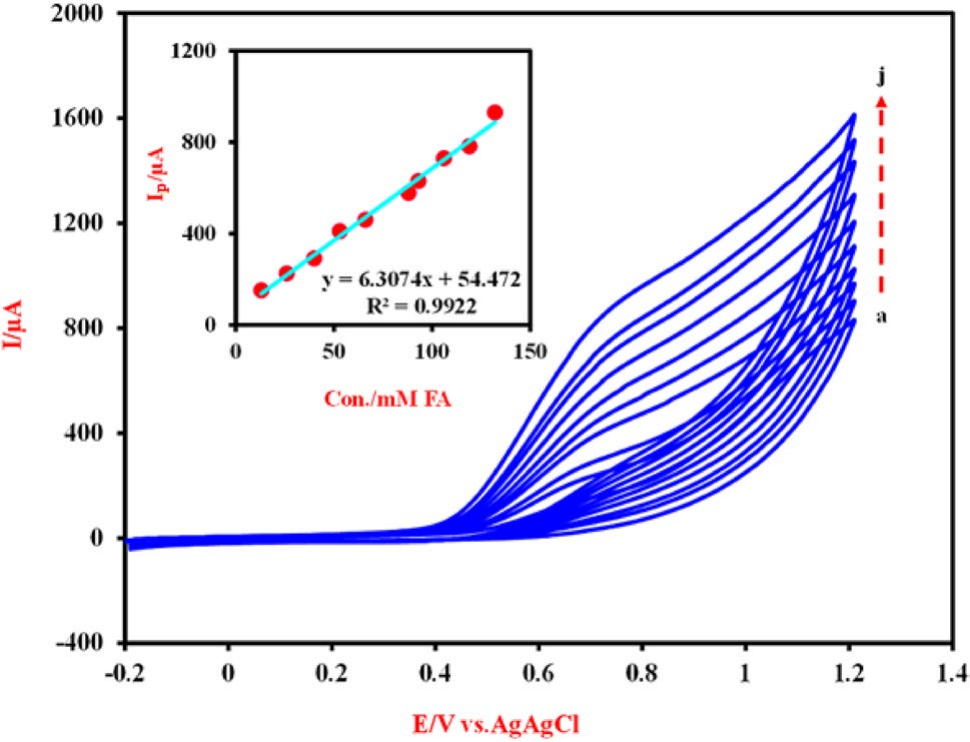
Cyclic voltammograms of the Cu-(PTA) MOFs/MgAl-LDH/CPE for electrocatalytic oxidation of FA at the scan rate of 50 mV s^-1^ in 0.1 M NaOH solution in different concentrations of FA: (**a**) 13, (**b**) 26, (**c**) 40, (**d**) 53 (**e**) 66, (**f**) 88, (**g**) 93, (**h**) 106, (**i**) 119 and (**j**) 132 mM, respectively. Inset is the oxidation peak current of FA vs the concentration of FA.

Furthermore, to investigate the relationship between the concentration of the background electrolyte solution and the anodic peak current of FA electrooxidation, the CVs of the Cu-(PTA) MOFs/MgAl-LDH were drawn in the presence of 66 mM FA and different concentrations of NaOH and the obtained results shown in Fig. 7B. It was observed that, (inset of Fig. 7B), with increasing the NaOH concentration from 0.025 to 0.1 M the anodic peak currents increase, but in the higher concentration of NaOH (> 0.1), the observed anodic peak current rapidly decreased. It seems that hydroxide ions compete with FA in occupying the sites of Cu-(PTA) MOFs/MgAl-LDH composite composition and reduces the active sites^75,78^. Therefore, the concentration of 0.1 M NaOH was chosen as the optimal concentration. Also, the CVs of the Cu-(PTA) MOFs/MgAl-LDH/CPE were investigated in 0.1 M NaOH solution containing different concentrations of FA in the potential range − 0.2 to 1.2 V (all CVs at scan rate of 50 mV s^-1^) and the obtained results shown in Fig. 8. Inset of Fig. 8 shows the oxidation peak current of FA vs the concentration of FA. As can be seen, the anodic peak current increases with increasing FA concentrations with linear correlation (R^2^ = 0.9922).

**Figure 9:**
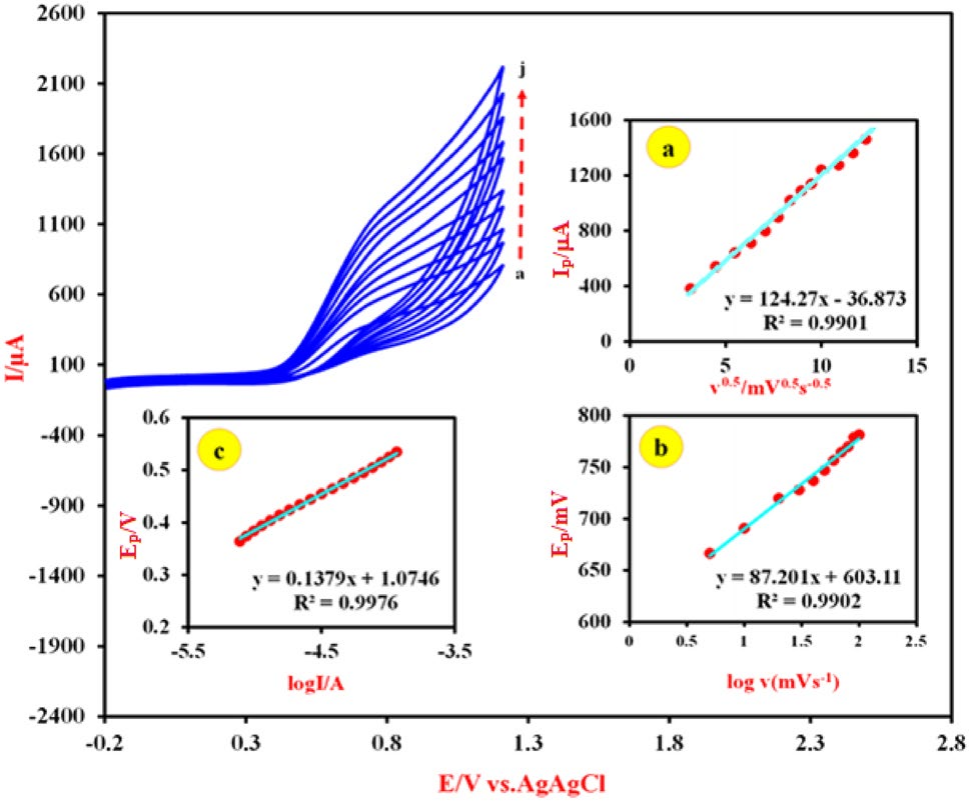
Cyclic voltammograms of the Cu-(PTA) MOFs/MgAl-LDH/CPE in 0.1 M NaOH containing 66 mM of FA at different scan rate (10–250 mV s^-1^). Inset (**a**): Plot of anodic peak currents with square root of scan rate, υ. Inset (**b**) the plot of E_p_ vs. Log υ. Inset (**c**) the Tafel plot for FA oxidation at the same electrode from the CV at a scan rate of 5 mV s^-1^.

By referring to reference electrochemistry books^79^, it can be said that the cyclic voltammetry technique could provide information about the charge transfer processes, electrode stability, and effect of chemical reactions on the electrode reactions. To determine the nature of the anodic peak current dependency on the electrocatalytic oxidation of FA, the CVs of the Cu-(PTA) MOFs/MgAl-LDH were recorded in the presence of 66 mM FA + 0.1 M NaOH in different scan rates (10–250 mVs) and the results shown in Fig. 9. As can be seen from the CVs, by increasing the scan rate of potential, the potential of the anodic peak current (E_p_) of FA shifts to more positive potentials, which indicates the existence of a kinetic limitation in the electrode reaction process between Cu-(PTA) MOFs/MgAl-LDH and FA^80^. Also, according to the information obtained from the data processing, drawn graphs and the plot of I_p_ vs. square root of scan rate (υ^1/2^) (Fig. 9 inset a), the anodic peak currents show linear dependency with the square root of scan rate. This behavior is the characteristic of a diffusion-controlled process (i.e. the spontaneous transfer of the electroactive species from regions of higher concentrations to regions of lower concentrations near surface of the electrode)^81^. The second CV segment is the E_p_ versus log υ (Fig. 9 inset b) and slope of dEp/dlog υ which was found to be 87.201, so b = 177.402. From the Eq. (9) and assuming one electron transfer in rate-determining step, n_α_ = 1, a charge transfer coefficient (α) of the FA oxidation was calculated as 0.61^82^:

9 $${E}_{P}=\frac{\mathrm{b\, log\, v}}{2}+{\text{constan}}$$

Also, from the Tafel plot (Fig. 9 inset c) at a low scan rate of 5 mVs^−1^, the value of the exchange current density (J°) was obtained as 23 mA cm^-2^.

**Figure 10:**
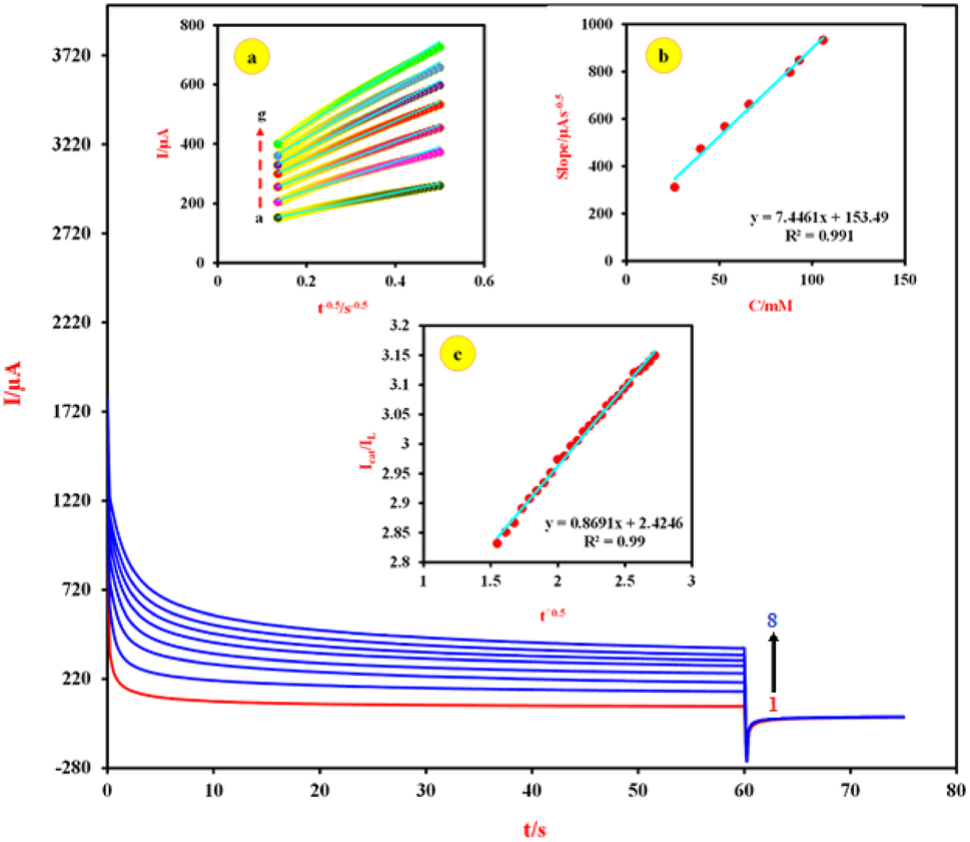
Chronoamperograms obtained at the Cu-(PTA) MOFs/MgAl-LDH/CPE (1) absence and presence of (2) 26, (3) 40, (4) 53, (5) 66, (6) 88, (7) 93 and (8) 106 mM of FA in 0.1 M NaOH solution. First and second potential steps were 0.0 and 0.74 V respectively. Inset (**a**): Dependence of current on t^-0.5^, derived from the data of chronoamperograms in the main panel. Inset (**b**): Plot of the slopes of the straight lines against the FA concentrations. Inset (**c**) dependence of I_cat_/I_L_ on t^0.5^, derived from the data of chronoamperograms.

Furthermore, chronoamperometry (CA) was used to measure the diffusion coefficient and catalytic rate constant of electrooxidation reaction FA at the Cu-(PTA) MOFs/MgAl-LDH and the obtained results shown in Fig. 10. Also, the chronoamperograms curves for various FA concentrations were recorded at the Cu-(PTA) MOFs/MgAl-LDH (Fig. 10 curve 1 in the absence of the FA and curves 2–8 in presence of FA at the concentration ranges of 26–106 mM, respectively). Value of diffusion coefficient was calculated from Cottrell equation and from the results in inset (a) of Fig. 10. The value of diffusion coefficient was found as 1.18 × 10^–6^ cm^2^s^-1^. On the other hand, the catalytic rate constant (k_cat_), for the electrooxidation reaction of FA at Cu-(PTA) MOFs/MgAl-LDH was obtained according to the Galus method and Eq. (10) ^75,82,83^:

10 $$\frac{{{\text{I}}}_{{\text{cat}}}}{{{\text{I}}}_{{\text{L}}}}={\upgamma }^{0.5 }{\uppi }^{0.5}={({{\text{K}}}_{{\text{cat}}}\mathrm{C\pi })}^{0.5}{{\text{t}}}^{0.5}$$

where I_cat_ and I_L_ are the currents at the modified electrode in the presence and absence of FA, respectively, and γ = kC_o_t [C_o_ is the bulk concentration of FA (mol cm^-3^)], k_cat_ catalytic rate constant (cm^3^ mol^-1^ s^-1^) and t is the time elapsed (s). Based on the plot of the slopes of the straight lines against the FA concentration (inset b of Fig. 10), dependence of I_cat_/I_L_ to the t^0.5^ (inset c of Fig. 10) and Eq. (10), the average value of k_cat_ is obtained as 0.4537 × 10^4^ cm^3^ mol^−1^ s^−1^^84–86^.

**Figure 11:**
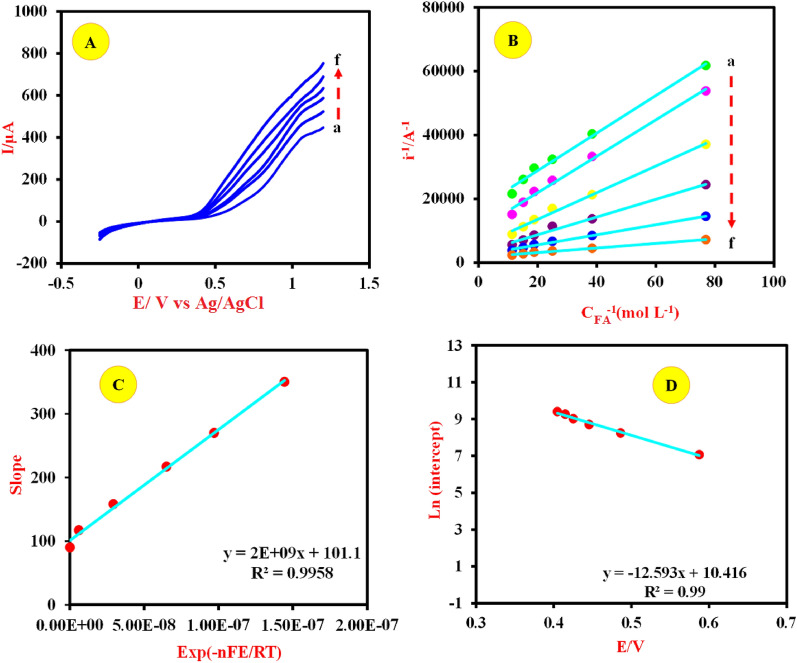
(**A**) Pseudo-steady state polarization curves of the Cu-(PTA) MOFs/MgAl-LDH/CPE obtained in (**a**) 13, (**b**) 26, (**c**) 40, (**d**) 53, (**e**) 66 and (**h**) 88 mM FA, respectively. The potential scan rate is 5 mVs^−1^ and rotation speed of the electrode = 3000 r/min. (**B**) Plot of i^-1^ against C_FA_^-1^ at various potentials: (**a**) 404.7, (**b**) 434.9, (**c**) 485.2, (**d**) 545.7, (**d**) 626.2, and (**h**) 817.6 mV as curves (**a**–**f**). (**C**) Plot of the slopes (of curves in B) vs. Exp (-nFE/RT). (**D**) Plot of the Ln (intercepts) (of curves in B) vs. applied potential.

The steady-state polarization curves for the electrooxidation of FA on the Cu-(PTA) MOFs/MgAl-LDH at different concentrations of FA in 0.1 M NaOH solution were recorded and presented in Fig. 11A. During the tests, to avoid the interference of mass transfer in the kinetics measurements rotation speed of the electrode was fixed at 3000 r/min. It can be seen that; the oxidation process begins around potential 404 mV (vs. Ag/AgCl) and reached a highest level at potential at 818 mV (vs. Ag/AgCl) while oxygen evolution begins at higher potential values. In the course of reaction, the coverage of Cu ^III^ increases and reaches a stable state level [Eq. (11)]^71,72^.

11 $${Cu}^{II }\begin{array}{c}\stackrel{{K}_{1}(E)}{\longrightarrow }\\ \underset{{K}_{-1}(E)}{\longleftarrow }\end{array}{Cu}^{III}+e$$

12 $$Cu\left(III\right)+C{H}_{2}O\left(FA\right)\stackrel{{K}_{2\left(E\right)}}{\longrightarrow }Cu\left(II\right)+Oxidation producs$$

In this case, the oxidation current based on the Eq. (12) can be calculated according to the following Eq. (13).

13 $$i=\left(\frac{{2FAK}_{1}\Gamma {K}_{2}{C}_{FA}}{{K}_{1}+{K}_{-1}+2{K}_{2}{C}_{FA}}\right)$$

14 $$[{K}_{1}\left(E\right)={K}_{1}^{0}exp\left[\frac{\alpha nFE}{RT}\right] and {K}_{-1}\left(E\right)={K}_{-1}^{0}exp\left[\frac{\left(\alpha -1\right)nFE}{RT}\right]]$$

Figure 11B demonstrate the plots of reverse of i against reverse of C_FA_ which obtained through the curve with a straight line at different potentials and Eq. (15) ^85–88^.

15 $${i}^{-1}={\left({FAK}_{1}\Gamma \right)}^{-1}+\left(\frac{{K}_{1}+{K}_{2}}{{2FAK}_{1}{\Gamma K}_{2}}\right){{C}_{FA}}^{-1}$$

It is noteworthy that both the slopes and intercepts in Fig. 11B are both dependent on the value of the potential. The slope of the graph was plotted against exp(-nFE/RT) with n = 1 and presented in Fig. 11C. By referring to this diagram and Eqs. (13, 14 and 15) the rate constant of reaction, k_1_Γ and ratio of k^0^_-1_/k^0^_1_ were calculated as 2.866 × 10^-9^ cm.s^-1^ and 1.97 × 10^–7^, respectively. In the following, the variation of the intercepts of the lines in Fig. 11C vs applied potential in a semi-log scale is shown in Fig. 11D. Using this graph and Eqs. (13, 14 and 15) the magnitude of k^0^_1_ was obtained as 2.47 × 10^–9^ mol cm^-2^ s^-1^.

#### Stability study of the Cu-(PTA) MOFs/MgAl-LDH/CPE

**Figure 12:**
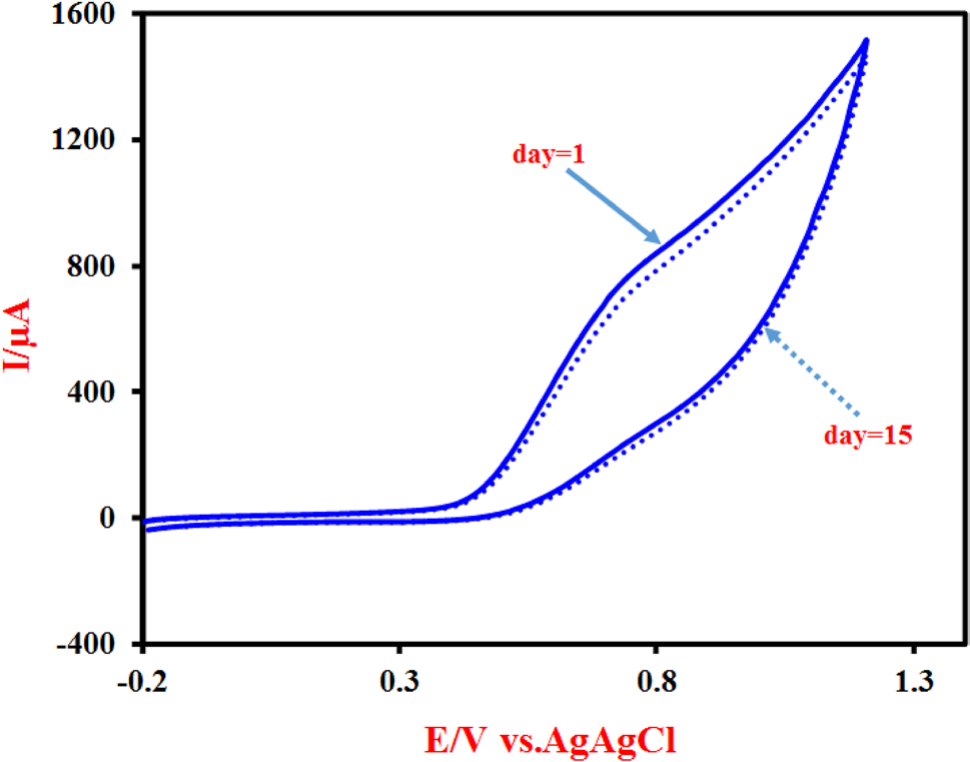
Stability test of the Cu-(PTA) MOFs/MgAl-LDH/CPE in 0.1 M NaOH + 0.12 M FA solution in the first day (day 1) and after long-term operation period (15 days) in various electrochemical methods (day 15).

The stability of the Cu-(PTA) MOFs/MgAl-LDH/CPE after a working applied course interval of 15 days (long-term operation period in various electrochemical methods) was investigated by recording the current response of FA oxidation in the same condition. The outcomes show that, negligible changes (< 5%), among the retained current response compared with the initial current as illustrated in Fig. 12. Actually, the changes in the electrocatalytic activity are negligible and the level of current remains nearly constant (CV_day1≈_CV_day15_). Considering that this material (Cu-(PTA) MOFs/MgAl-LDH) was able to show approximately constant electrocatalytic activity toward the electrooxidation of FA after fifteen days and performing various electrochemical tests during these days at the worked pH (Fig. 12), can indicates that this material (nanocomposite) has sufficient stability in these conditions. In other words, its structure has not changed during these fifteen days in the worked pH solution and various electrochemical tests. Otherwise, its electrocatalytic activity should be reduced or lost. These results demonstrated that the Cu-(PTA) MOFs/MgAl-LDH/CPE has a promising potential as a stable and efficient electrocatalyst for oxidation of FA under the optimized experimental condition. Finally, the electrochemical performance of the Cu-(PTA) MOFs/MgAl-LDH/CPE as an efficient electrocatalyst for FA electrooxidation was compared with other reported electrocatalysts and shown in Table 2. The data in Table 2 shows that the electrocatalytic performance of the Cu-(PTA) MOFs/MgAl-LDH/CPE is superior or comparable with most reported electrocatalysts toward FA electrooxidation reactions.

**Table 2:** Comparison of the electron-transfer coefficient (α), diffusion coefficient (D_FA_), exchange current density (J°) and catalytic rate constant (k_cal_) of different modified electrodes used in electrocatalytic oxidation of FA.

Modified electrodes	α	D_FA (_cm^2^ s^−1^)	J° (mA cm^-2^)	K_cal_ (cm^3^mol^–1^ s^–1^)	Ref
Ni-CHIT/CPE^a^	0.47	2.68 × 10 − 6	–	2.06 × 105	^73^
Copper electrode	0.66	-	–	4.6 × 10^1^	^80^
Ni(OH)_2_-Beta/CPE	0.69	4.4 × 10^−7^	–	2.08 × 10^6^	^81^
nano-CuO/GCE^b^	0.56	–	–	2.5 × 10^7^	^82^
NiWO_4_-NPs/CPE	069	40.4 × 10 − 4	–	1.37 × 10^4^	^84^
Cu/P(2ADPA)/MCPE	–	–	25.56	7.16 × 10^6^	^85^
Ni/P-1,5-DAN/MCPE	–	–	7	2 × 10^6^	^86^
Ni-ZSM-5/CPE	0.49	8.575 × 10^–6^	13.02	9.064 × 10^3^	^88^
Cu-(PTA) MOFs/MgAl-LDH/CPE	0.61	1.18 × 10^–6^	23	4.537 × 10^3^	This work

^a^CPE, Carbon Paste Electrode.

^b^GCE, Glassy Carbon Electrode.

## Conclusions

The main objective of this study was the synthesis of Cu-(PTA) MOFs/MgAl-LDH via a simple electrocoagulation (EC) process and chemical method at room temperature for electrocatalysis proposes. The physicochemical characterizations of the synthesized electrocatalysts were investigated by different techniques: SEM, EDX, TEM, PSD, BET, TGA, XRD, FT-IR, DLS, and etc. The Cu-(PTA) MOFs/MgAl-LDH nanocomposite was used to modification of the carbon paste electrode (CPE); Cu-(PTA) MOFs/MgAl-LDH/CPE. The electrochemical performance of the Cu-(PTA) MOFs/MgAl-LDH/CPE was studied through the utilization of electrochemical methods. The electrochemical studies show that the Cu-(PTA) MOFs/MgAl-LDH/CPE has high electrocatalytic activity toward FA oxidation in basic media. The results show that the oxidation peak of FA increases with increasing the FA concentration and electrooxidation of FA is under a diffusion-controlled process. In addition, based on the obtained data, the good electrochemical properties of the Cu-(PTA) MOFs/MgAl-LDH/CPE has been revealed for the estimation of the important electrochemical parameters including [electron-transfer coefficient (α), diffusion coefficient (D_FA_), exchange current density (J°) and catalytic rate constant (k_cal_) and the ratio of k_0-1_/k_01_] in the electrooxidation of FA. Finally, in conclusion, the proposed Cu-(PTA) MOFs/MgAl-LDH/CPE with high stability and excellent properties can be expected to have applications in the field of fuel cells and electrochemical sensors.

## Data Availability

The data that support the findings of this study are available from the corresponding author upon request.
